# Study on Risk Assessment Methods and Zoning of Hazardous Chemicals Leaking into Seas

**DOI:** 10.3390/ijerph192214713

**Published:** 2022-11-09

**Authors:** Jiangyue Wu, Guodong Xu, Haoshuang Guo, Yao Zhang, Fang Xia, Gang Fang

**Affiliations:** 1 National Marine Hazard Mitigation Service, Ministry of Natural Resources of the People’s Republic of China, Beijing 100194, China; 2 School of Land Science and Technology, China University of Geosciences, Beijing 100083, China; 3 Chinese Research Academy of Environmental Sciences, Beijing 100012, China

**Keywords:** hazardous chemicals, leak into seas, risk assessment, zoning

## Abstract

In China, studies on the regional risk assessment of hazardous chemicals have been carried out for only a few years, and there are few studies on hazardous chemicals leaking into seas. Previous regional-risk-assessment methods considered a single risk factor for most assessment targets, and comprehensive considerations of risk sources and sensitive resources for a study area are not sufficiently included. Based on previous work, this study established a regional-risk-assessment method for hazardous chemicals leaking into seas. This method considered the hazards of hazardous chemicals and the tolerance of the regional environment by means of a case study in Tianjin. The results showed that the risk level of the enterprise was Grade I, classified as a high-risk source of hazardous chemicals; the main reasons were the strong toxicity and large quantity of hazardous chemicals. This method provides technical support for scientifically assessing marine-environmental-risk levels for hazardous-chemical-leakage areas and for carrying out risk-prevention and restoration assessments of hazardous chemicals leaking into seas.

## 1. Introduction

The chemical industry has gradually become the leading industry in China. While the use of many chemicals improves production quality and quality of life, their inherently dangerous properties also pose a great threat to human health and the natural environment [1]. At the same time, the coastal layout of a large number of chemical parks, run by enterprises who engage in large-scale hazardous chemical production, storage and transportation in coastal zones, has also aggravated the risk of hazardous chemicals leaking into the sea. The import and export routes of hazardous-chemical raw materials are mainly ports and fairways [2], and with the expansion of port throughput and the increase in the frequency of ship transportation, the probability of accidents such as the leakage of hazardous chemicals at sea increases [3]. On 20 March 2018, the Central Committee of the Communist Party of China issued the Plan for Deepening the Reform of Party and State Institutions [4], which clarified that the main responsibilities of the newly formed Ministry of Natural Resources are to “uniformly exercise the responsibilities of owners of all natural resource assets owned by the whole people, and uniformly exercise the responsibilities of controlling the use of all land and space and ecological protection and restoration”. Therefore, carrying out risk assessments in areas where hazardous chemicals have spilled into the sea is of great significance for improving responses to marine disasters, territorial spatial planning and use control, and improving the ability to respond to marine environmental disasters and protecting resources.

In the 1960s and 1970s, developed countries enacted laws on the management of chemical substances, which prompted United Nations agencies to gradually establish and implement relevant international conventions [5]. Initially, developed countries mainly identified the hazards of chemicals to achieve safety management [6]. In the 1990s, risk-assessment techniques gradually developed, and chemical management in developed countries began to shift to a form of risk management that comprehensively considered the inherent hazards of chemicals and their exposures [7], that is, risk assessment using scientific procedures, and then further analyzed the benefits of chemicals to society, their impact on socioeconomic development, and alternative technologies with which to make risk-management decisions. In the process of chemical-risk management, chemical-risk-assessment technology is the core technical means of chemical management. To cooperate with chemical management, international organizations and developed countries have successively issued guidelines or norms to guide risk assessment [8].

Research outside China on the risk assessment and prediction of hazardous chemical accidents began earlier, and it is more systematic in both theory and method. In terms of policy, many developed countries have incorporated environmental risk assessment into the scope of environmental management, with policies such as the guidelines on “Controlling Accidents Affecting People inside and Outside Factories and Major Environmental Hazards” issued by the Department of Environment and Science of the World Bank in 1985; the European Union (EU) legislation from 1987, which stipulates that environmental risk assessments must be carried out for factories that may be at risk of chemical accidents; and the Appel Plan (APELL), developed in 1988 by the United Nations Environment Programme (UNEP), which is used to deal with environmental pollution accidents that are difficult to prevent and may cause serious harm to human health and the ecological environment [9].

In 1996, the EU published the first edition of the Technical Guidelines for Risk Assessment (TGRA), applicable to various chemicals [10], which detailed the technical elements of chemical-risk assessment. Overall, the EU’s chemical-risk assessment focuses on the integrated management of chemicals, and it is the world’s leading approach to chemical-risk management. Chemical-management and risk-assessment technology in the United States originated in the Pollution Prevention Act implemented by the U.S. Environmental Protection Agency (EPA) Office of Pollution Prevention and Toxic Substances in 1976; the relevant model tools for chemical-risk assessment in the U.S. are the evaluation of chemicals’ physicochemical properties and environmental mapping models, predictive hazard and toxicity models, and emission, exposure, and risk models.

BP International, a well-known British multinational company, absorbed the methodology of major-accident-risk management used by multinational oil companies and formed a set of major accident risk-management processes [11]. Marhavilas et al. [12] established an assessment framework based on the traditional risk-assessment framework, applying qualitative assessment and quantitative assessment by using deterministic methods and uncertain methods, respectively. The National Environmental Exposure Laboratory of the U.S. EPA proposed the Framework for Risk Analysis in Multimedia Environmental Systems–Multimedia, Multipathway, and Multireceptor Risk Assessment (FRAMES–3MRA) [13]. Romer et al. [14] proposed the natural-accident-consequence-description method (covering contaminated coastal areas, the number of dead birds and fish, etc.) to establish a corresponding risk-assessment model for hazardous-chemical-water-transport accidents and innovatively improved the evaluation of the consequences for living organisms in the model. Wessberg et al. [15] estimated risk by constructing activity and process models that analyze the probability and consequences of an accident. Matthiessen et al. [16] studied the environmental hazards of hazardous chemicals from a unique perspective, that is, by measuring the environmental impact of hazardous chemicals by observing hormonal interference in living organisms, and used this as a basis for a corresponding risk assessment. Zhang et al. [17] used GIS technology to assess the threat of chemical accidents to people and the environment, established a risk-index model, and, with the support of chemical characteristics and environmental-resource-information databases, studied the effects of local pollution from chemical accidents on human health, groundwater, surface water, and soil resources, and provided relevant decision-making support for managers.

The research status of risk-assessment technology used for hazardous chemicals in China can be divided into the following aspects:

(1) Theoretical research on risk assessment of hazardous chemicals. At present, risk-assessment research on major sources of hazardous chemicals mainly focuses on four aspects: multiobjective fuzzy theory [18], grey theory [19,20], the domino effect [21], and variable fuzzy set theory [22].
(2) Qualitative-risk-assessment technology for hazardous chemicals. In March 2013, the Ministry of Environmental Protection issued the Guidelines for the Preparation of Environmental Risk Assessment Reports for Hazardous Chemicals under Key Environmental Management (Trial Implementation) [23], and in April 2014, it issued the Guidelines for Risk Assessment of Environmental Emergencies in Enterprises (Trial Implementation) [24]. The two specifications took hazardous chemicals and key enterprises as their respective starting points and established specific methods for environmental-risk-level assessment, namely, environmental risk assessment of hazardous chemicals and risk assessment of environmental emergencies. In addition, Liu Zhiguo et al. [25], on the basis of the analysis of the risk characteristics of coastal-chemical-risk sources, constructed a comprehensive assessment index system for the environmental risk of chemical risk sources based on risk sources, control mechanisms, and risk receptors, established a corresponding risk-evaluation model, and proposed a three-level risk-management system. The risk-assessment method adopted by Wang Shouyun [26] first determines the sea area where dangerous goods leakage accidents may occur, assesses the frequency of accidents, and then manages the risks in different geographical areas.
(3) Quantitative risk-assessment technology for hazardous chemicals. Li Qiujin et al. [27] conducted a study on a series of accidents caused by chlorine leakage, analyzed the possible accident scenarios according to the quantitative-risk-evaluation procedure of the given accident situation, discussed and analyzed each accident situation separately, and finally focused on the highest-risk scenario. Zhao Wenfang and Wu Zhifeng [28] determined the method of equipment unit selection and the statistical scope of environmental information data, proposed the calculation method and implementation method of accident frequency, accident consequences, personal risk, social risk, etc., and established a highly practical technical scheme for the quantitative risk assessment of major sources of hazardous chemicals. Deng Qigen et al. [29] analyzed the mathematical model of the accident probability of chemical enterprises on the basis of the Markov accident-probability-hypothesis model. Chen Guohua et al. [30], based on the basic principle of quantitative risk evaluation, proposed a regional risk-assessment method, and the quantitative evaluation results describing the overall risk status of the region were obtained by applying the superposition principle.
(4) The innovative application of computer technology in the risk assessment of major sources of hazardous chemicals. In terms of the risk assessment of major hazards in petrochemical enterprises, Liang Chenghao and Lü Dong [31] used the programming language Visual Basic to establish a petrochemical-enterprise fire-and-explosion risk-assessment system that can be used for the risk assessment of petrochemical enterprises by calling and controlling the Oracle Database.

In summary, regional-risk-assessment research on hazardous chemicals in China started late, and there has been less research on the methods for assessing the leakage of hazardous chemicals into the sea. In the existing regional-risk-assessment methodology, most of the assessment objects are single risk factors, and there is a deficiency in that the comprehensive impact of other risk sources and sensitive resources in the study area is not fully considered.

In this study, to carry out the risk assessment and zoning of hazardous chemicals leaking into seas, we developed a regional-risk-assessment index system for hazardous chemicals spilling into the sea, focusing on the whole coastal area of China.

## 2. Methods

**Figure 1:**
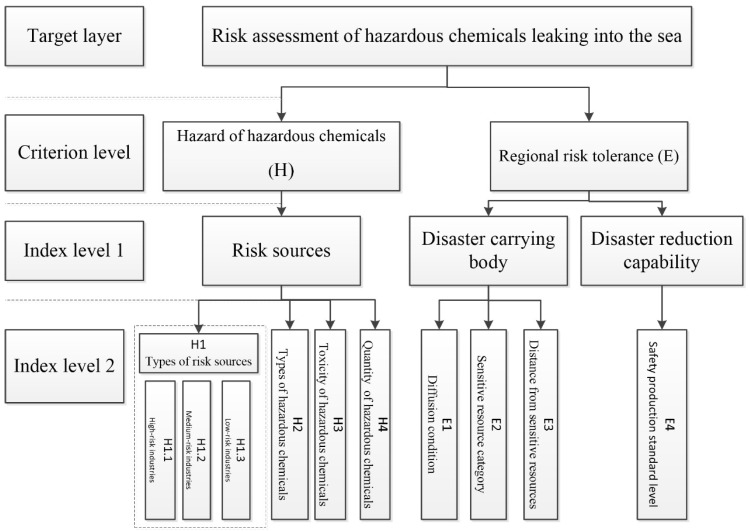
Risk-assessment indicators for hazardous chemicals leaking into the sea.

Due to the insufficient accumulation of risk data in the environmental field in China, it is difficult to scientifically define the risk level of each evaluation index. Therefore, by comprehensively drawing on the grading methods from previous research at home and abroad, based on the responsibilities of the Ministry of Natural Resources, this study initially established a risk-grading index system (Figure 1) that includes 8 specific indicators of 2 criterion layers (hazard and regional risk tolerance of hazardous chemicals) and used the scoring method to determine the risk level of each indicator through a literature review, data collection, and expert consultation.

### 2.1. Risk-Assessment Methodology

Considering that environmental risks may arise from defects in any link in the risk system, the comprehensive evaluation of risk zoning adopts a combined algorithm of weighted summation and weighted multiplication. In the calculation of the criterion layer from specific indicators, different specific indicators represent different aspects of the same risk factor, and the weighted summation method is used to calculate the criterion layer following Equation (1). In addition, the analytic hierarchy process (AHP) and Delphi method (Delphi) are used to determine the weights, the weight coefficients between the criterion layer and each specific indicator are then determined, and the sum of the coefficients is maintained as 1 when determining the weights between specific indicators.

(1) $$R=\sum_{i=1}^{n}C_{i}S_{i}$$

where *R* is the regional-risk-assessment score, *C_i_* is the weight value of the specific indicator, and *S_i_* is the score of the indicator.

### 2.2. Indicator System

#### 2.2.1. Hazardous Chemicals

The hazard assessment of hazardous chemicals should comprehensively consider the types of risk sources involved in hazardous chemicals, the types of hazardous chemicals, and the quantity of hazardous chemicals. The details are as follows:

H1. Risk-source type: The probability of environmental accidents in various industry types is different, and we devided into three types: high-risk-industry types (H1. 1), medium-risk-industry types (H1. 2) and low-risk industry types (H1. 3). High-risk-industry types include chemical raw materials and chemical manufacturing, crude-oil processing and petroleum-product manufacturing; medium-risk-industry types include medicine, printing and dyeing, coatings, metal surface treatment and hot processing; low-risk industry types include steelmaking and steel rolling.

H2. Hazardous chemical types: Different types of hazardous chemicals have different degrees of impact on the environment, and internationally, the European Standard Behaviour Classification System (SEBC code; Bonn Agreement, 1991) is generally used to classify the physical behavior of chemicals after entering the sea [32]. According to SEBC, once some hazardous chemicals leak into sea, there four situations occur: floating (F), dissolution (D), sedimentation (S), and volatilization (E). Furthermore, since some hazardous chemicals show multiple physical behaviors, SEBC classified hazardous chemicals into 8 categories: floating volatilization (FE), rapid dissolution volatilization (DE), rapid dissolution (D), floating (F), floating dissolution (FD), floating volatilization and dissolution (FED), sedimentation (S), and sedimentation dissolution (SD). Volatile hazardous chemicals (FE, DE, and FED) volatilize in the air, which causes relatively little harm to the marine environment. Sedimentary hazardous chemicals (S and SD) are easy to salvage and dispose of, which causes relatively moderate harm to the marine environment. Thus, considering the different hazards of different types of hazardous chemicals after they leak into the sea, they are divided into three levels: high (D, F, FD), medium (S, SD), and low (FE, DE, FED).

H3. Toxicity of hazardous chemicals: The Joint Group of Experts on the Scientific Aspects of Marine Pollution (GESAMP) comprehensively considers the bioaccumulability, stability, and aquatic biological toxicity of hazardous chemicals and divides the ecotoxicity of hazardous chemicals into grades 1 to 6 [33,34,35,36,37,38] (Table 1). Drawing on this reference, to improve the operability and applicability of the method, the grading scale was divided into three levels: high (levels 5 to 6), medium (levels 3 to 4), and low (levels 1 to 2).

H4. Quantity of hazardous chemicals: Refers to the number of hazardous chemicals used, stored, and produced in the region. Based on the division of the “List of Hazardous Substances and Critical Quantities of Environmental Emergencies” of the Ministry in Environmental Protection’s “Guidelines for Risk Assessment of Environmental Emergencies in Enterprises (Trial),” considering the critical amount of hazardous chemicals in the region, it is divided into three levels: high, medium, and low.

#### 2.2.2. Regional Risk Tolerance

Regional risk tolerance is divided into two factors: disaster bearer and disaster-mitigation capacity. Disaster bearer comprehensively considers the background values of characteristic pollutants in the marine environment, the impact of topography and geomorphology, the connection to the ocean, the category of sensitive resources, and the distance from sensitive resources. Disaster-reduction capacities should take into account regional-risk-control capacities. The specific instructions are as follows:

E1. Diffusion condition: Refers to the drift diffusion of hazardous chemicals after they enter the sea; the wider the scope of the diffusion, the greater the degree of its impact on the marine environment. The spread situation is divided into open sea, semi-enclosed bay, and harbor pool, and its impact on the marine environment is open sea > semi-enclosed bay > port pool.

E2. Sensitive-resources category: With reference to the marine-water-quality standard [34], sensitive resources are divided into four categories: the first category is marine fishery waters, marine nature reserves and rare- and endangered-marine-life reserves; the second category is aquaculture areas, beaches, and marine sports or recreation areas, where the human body is in direct contact with seawater and industrial water areas directly related to human consumption; the third category is general industrial water areas and coastal-scenic-tourism areas; and the fourth category is marine-port water areas and marine-development-operation areas.

E3. Distance from sensitive resources: The distance from sensitive resources reflects the degree of damage caused by environmental accidents. After a spatial analysis of environmental risk sources and surrounding sensitive points through GIS, the distance from the most direct leakage point to the nearest environmentally sensitive point according to the risk source is divided into 3 levels: <3 km, 3 to 10 km, and ≥10 km.

E4. Enterprise safety-production-standardization level: According to the provisions of the Administrative Measures for the Evaluation of Enterprise Safety Production Standardization (Trial) [35], the safety-production standardization of hazardous chemical enterprises is divided into first-level, second-level, and third-level. The first-level enterprises are reviewed and announced by the State Administration of Work Safety; the second-level enterprises are reviewed and announced by the safety-production supervision and management department of the province (autonomous region or municipality directly under the Central Government) in which the enterprise is located and the Xinjiang Production and Construction Corps; and third-level enterprises are reviewed and announced by the safety-production supervision and management department of the district city (prefecture, league) in which the enterprise is located.

### 2.3. Classification Values

To make the weights determined more representative and maximize the sample size, the relevant experts of the First Institute of Oceanography of the State Oceanic Administration, Xiamen University, Beijing Normal University, Beijing University of Chemical Technology, Dalian Maritime University, CNOOC Safety Technical Service Company, Beihai Environmental Monitoring Center of the State Oceanic Administration, and other units were invited to comprehensively construct a judgement matrix of expert scores to determine the relative importance of weights of each layer of indicators (Table 2).

#### 2.3.1. Classification of Hazardous Chemicals

According to the hazard scores of hazardous chemicals, the hazards of hazardous chemicals were divided into four levels (Table 3).

#### 2.3.2. Classification of Regional Risk Tolerance

According to the regional-risk-tolerance scores, the regional risk tolerance was divided into four levels (Table 4).

### 2.4. Classification Method

According to China’s current administrative management system, with the county (district) as the unit, a regional risk assessment was carried out, and the assessment scope was 10 km on the seaward side of the coastline and, on the landward side, up to the maximum-high-tide line, focusing on the risk sources for sources of dangerous chemical leakage into the sea, such as hazardous-chemical enterprises located within 1 km of the coastline on the landward side or adjacent to rivers entering the sea. The risk-assessment-level relationship of dangerous chemicals leaking into the sea area is shown in Table 5.

## 3. Case Application

To test the applicability of the risk-source-assessment-index system, a typical enterprise in Tianjin Binhai New Area was selected as a research case for risk-source-level assessment. First, basic information, such as the geographical location coordinates, the types and quantities of hazardous chemicals involved, and the level of the enterprise’s safety-production standards, was collected and sorted. Second, according to the “Tianjin Marine Functional Zoning” (2011–2020), the conditions for the potential diffusion of chemicals into the sea from the company, the types of sensitive resources nearby, and their distance from sensitive resources were determined. Finally, the scores for each risk factor were calculated according to the established risk-assessment method for the leakage of hazardous chemicals into the sea (Table 6). The company’s hazardous-chemical score was 26 points (Level I) and the regional risk tolerance was 17 points (Level II). The results showed that the risk level of the enterprise was Grade I, classified as a high-risk source of hazardous chemicals; the main reasons were the strong toxicity and large quantity of hazardous chemicals. Considering that this method is still under study, XX is used here instead of the actual company name.

## 4. Conclusions

By applying the recent research conducted at home and abroad to comprehensively consider the hazard and regional risk tolerance of hazardous chemicals, this study established a regional-risk-assessment-index system for hazardous chemicals spilling into the sea, including the type of risk source, the types of hazardous chemicals, the quantity of hazardous chemicals, the toxicity of hazardous chemicals, the diffusion conditions, the types of sensitive resources, the distance from sensitive resources, and the level of enterprise-safety-production standards. At the same time, a combination of the AHP and the Delphi method was used to determine the weights of the indicators, and comprehensive expert scores were assigned to the indicators. The index system not only takes into account the scientific rationality of index-setting and weight assignment, but also considers the applicability of the operationalization of risk assessment in the area in which hazardous chemicals are leaked into the sea. Unquestionably, there are still deficiencies in the risk assessment of hazardous chemicals leaking into the sea. The indicator system needs to be further adjusted and screened, the weight assignment needs to be further optimized and improved, and it is still difficult to obtain some indicators, such as the quantity of hazardous chemicals and the level of enterprise-safety-production standards.

In the next step, we will select the Bohai Sea area, carry out the application of the risk-assessment methods for hazardous chemicals leaking into the sea, further improve the assessment method and indicator system, and provide good technical support for the comprehensive management of the Bohai Sea.

## Figures and Tables

**Table 1:** Grading standard for ecological toxicities of dangerous chemicals of GESAMP.

Grading	Bioaccumulation			Stability	Aquatic Toxicity/(mg·L^−1^)			
	Log*K*_ow_	BCF	Description		Acute Toxicity		Chronic Toxicity	
					LC/EC_50_	Description	NOEC	Description
1	<1	Not available	No	Totally stable	>1000	Nontoxic	>1	No
2	1 ≤ - < 2	1 ≤ - < 10	Extremely low	Stable	100 < - ≤ 1000	Generally nontoxic	0.1 < - ≤ 1	Extremely low
3	2 ≤ - < 3	10≤ - < 100	Low	Low instability	10 < - ≤ 100	Low toxicity	0.01 < - ≤ 0.1	Low
4	3 ≤ - < 4	100 ≤ - < 500	Moderate	Moderate instability	1 < - ≤ 10	Moderate toxicity	0.001 < - ≤ 0.01	Moderate
5	4 ≤ - < 5	500 ≤ - < 4000	High	High instability	0.01 < - ≤ 1	High toxicity	≤0.001	High
6	5≤	4000≤	Extremely high	Extreme instability	0.01<	Extremely high toxicity		Extremely high

Note: BCF refers to the bioconcentration factor.

**Table 2:** Classification values for risk assessment of hazardous chemicals leaking into the sea.

Criterion Level	Index Level 1	Index Level 2	Index Refinement	Values
Hazard of hazardous chemicals H (0.5)	Risk sources	Types of risk sources H1 (0.25)	High environmental risk	3
			Moderate environmental risk	2
			Low environmental risk	1
		Types of hazardous chemicals H2 (0.25)	High	5
			Moderate	3
			Low	1
		Toxicity of hazardous chemicals H3 (0.25)	High	9
			Moderate	6
			Low	3
		Quantity of hazardous chemicals H4 (0.25)	>80%	9
			40~80%	6
			<40%	3
Regional risk tolerance E (0.5)	Disaster-carrying body (0.75)	Diffusion condition E1 (0.25)	Open sea area	3
			Semi-enclosed bay area	2
			Harbour basin	1
		Sensitive resource category E2 (0.25)	Marine fishery waters, marine nature reserves, and rare-and-endangered-marine-life reserves	9
			Aquaculture areas, bathing beaches, marine sports or recreational areas where the human body is in direct contact with sea water, and industrial water areas directly related to human consumption	5
			General industrial water area, coastal-scenic-tourist area	3
			Harbor basin, marine-development-operation area	1
		Distance from sensitive resources E3 (0.25)	<3 km	5
			3~10 km	3
			≥10 km	1
	Disaster-reduction capability (0.25)	Safety-production-standard level E4 (0.25)	Poor	9
			Ordinary	6
			Good	3

**Table 3:** Classification of hazardous chemicals.

Hazard Score of Hazardous Chemicals (H)	Classification
21 ≤ H ≤ 26	I
16 ≤ H < 21	II
12 ≤ H < 16	III
8 ≤ H < 12	IV

**Table 4:** Classification of regional risk tolerance.

Regional Risk Tolerance Score (E)	Classification
21 ≤ E ≤ 26	I
16 ≤ E < 21	II
11 ≤ E < 16	III
6 ≤ E < 11	IV

**Table 5:** Risk-assessment-level relationship of dangerous chemicals leaking into the sea area.

Regional Risk Tolerance					
Hazard of hazardous chemicals		Extremely low (IV)	Low (III)	High (II)	Extremely high (I)
	Extremely low (IV)	Extremely low (IV)	Extremely low (IV)	Low (III)	Low (III)
	Low (III)	Extremely low (IV)	Low (III)	High (II)	High (II)
	High (II)	Low (III)	High (II)	High (II)	Extremely high (I)
	Extremely high (I)	Low (III)	High (II)	Extremely high (I)	Extremely high (I)

**Table 6:** Risk-assessment level of XX company in Tianjin.

Criterion Level	Index Level 1	Index Level 2	Index Refinement	Values
Hazard of hazardous chemicals H (0.5)	Risk sources	Types of risk sources H1 (0.25)	High environmental risk	3
		Types of hazardous chemicals H2 (0.25)	High	5
		Toxicity of hazardous chemicals H3 (0.25)	High	9
		Quantity of hazardous chemicals H4 (0.25)	>80%	9
	Total			26
Regional risk tolerance E (0.5)	Disaster-carrying body (0.25)	Diffusion condition E1 (0.25)	Semi-enclosed bay area	2
		Sensitive resource category E2 (0.25)	Harbor basin, marine development operation area	1
		Distance from sensitive resources E3 (0.25)	<3 km	5
	Disaster-reduction capability (0.25)	Safety-production-standard level E4 (0.25)	Poor	9
	Total			17
Risk level				I

## Data Availability

Data available on request due to privacy and ethical restrictions.
